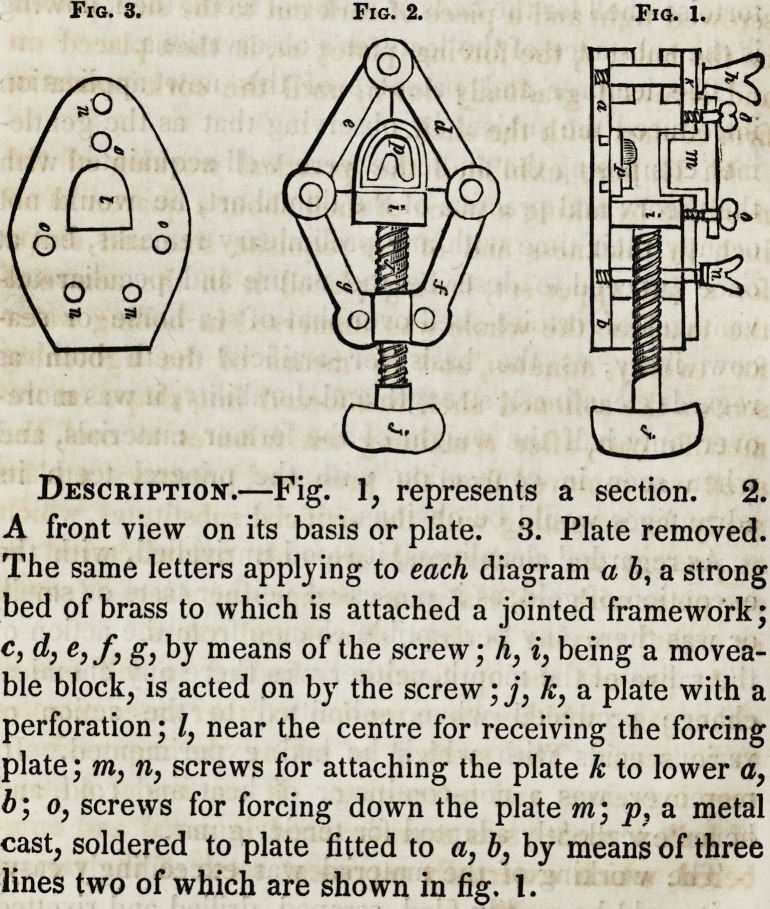# The Application of Tortoise-Shell to Mechanical Dentistry.—Reported for the Journal

**Published:** 1850-04

**Authors:** James Robinson


### ARTICLE VII.

*The application of Tortoise-shell to Mechanical Dentistry.—Reported for the Journal.* By Dr. James Robinson.

A large number of the dental profession attended by invitation at the Western Literary Institution, Leicester Square, London, on Tuesday, the 5th February, 1850, to hear a lecture by Mr. G. F. Harrington, Dentist of Portsmouth, on the application of tortoise-shell for the beds of artificial teeth, the following is the substance of the lecturer's observations.

The lecturer and patentee of this new application introduced the subject by observing that as the gentlemen composing the audience were well acquainted with the theory and practice of the dental art, he would not occupy their time with any preliminary remarks, but at once proceed to describe the nature and peculiar advantages of tortoise-shell over that of sea-horse or sea-cow ivory, as the basis for artificial teeth both as regards cleanliness, strength and durability, it was moreover only half the weight of the former materials, and when seen in connection with the mineral teeth its advantages would be obvious.

As regarded cleanliness, it stood unrivalled, with the exception of gold, as it possessed neither taste or smell, or was there any perceptible change from the action of the saliva of the mouth, neither was there any chemical change produced when submitted to the action of various acids with which he had experimented. It, moreover, was a non-conductor of heat and cold, and hence excellently adapted for tender gums.

The working of the material was exceedingly easy, as it could be readily filed, scraped, drilled and rivetted without the least danger of accident; it was non-absorbent and capable of receiving and retaining a high polish, and from the results of Mr. H's experiments for five years, he assured the meeting that it would be found more durable than either of the ivorys usually employed and probably equal to gold in that respect.

The lecturer then described a very ingenious piece of mechanism for fixing the shell into the requisite form. The following diagram represents the machine as far as we could understand.

Fig. 3.

Fig. 2.

Fig. 1.

Description.—Fig. 1, represents a section. 2. A front view on its basis or plate. 3. Plate removed. The same letters applying to *each* diagram *a b*, a strong bed of brass to which is attached a jointed framework; *c, d, e, f, g*, by means of the screw; *h, i*, being a moveable block, is acted on by the screw; *j, k*, a plate with a perforation; *l*, near the centre for receiving the forcing plate; *m, n*, screws for attaching the plate *k* to lower *a, b*; *o*, screws for forcing down the plate *m*; *p*, a metal cast, soldered to plate fitted to *a, b*, by means of three lines two of which are shown in fig. 1.

Action.—In using the machine, the screw *j*, is undone; the block *t*, moved back and the framework *c*, *d*, *e*, *f*, *g*, pressed so that the ends, *c* and *f*, *g*, approach, and the sides *d*, *e*, receive the horse-shoe sloped centre which will be found to have separated into three parts, viz: the end *t*, and the two sides *d*, *e*. The model plate *p*, is inserted in its place and a piece of tortoise-shell cut to the proper size and slope placed upon it; the screw *j*, is then turned gradually, when it will be found the horse-shoe opening has contracted; the plate *k*, is next screwed tight and a piece of cork cut to the size, placed in the hole; *l*, the forcing plate; *m*, is then placed on and screwed gradually down, until the cork comes in firm contact with the shell.

M. H. here exhibited the practical working of the machine by taking a piece of shell about a quarter of an inch in thickness and of the requisite size and shape for a full upper set, having placed it in the centre of the machine the whole was immersed in boiling water for twenty minutes, and by means of the tightening screws the softened shell was forced into shape on the metallic cast, after remaining ten minutes to cool, the shell was removed from the press and presented a plate ready for mounting with the artificial substitutes, which at present we understand are only manufactured in one piece with artificial gums, comprising a series (seven or eight) varying in sizes, shapes and colors.

At present the application is limited to full sets in either jaw; whither its extension to other cases remains to be seen. The method of taking the impression is very ingenious and interesting. It consists of a series of hollow model sets manufactured in metal and numbered, which externally represent teeth of the various width and depth usually required; this tray is filled with wax and pressed upon the gum to the proper depth in the usual manner. The patient is now desired to close the mouth which gives the requisite length, &c. By this means the lecturer stated the model, bite and adaptation in complete sets could be finished at one interview with the patient.

The operator has now to submit his model to a graduated guage invented for the purpose of taking the depth and width of his artificial substitutes, and as before observed a number of sizes and shapes are kept ready manufactured by Messrs. T. Ash & Co., corresponding to the number of his tray, by means of which he took the impression in the first instance, after selecting the one adapted to the case, it is mounted upon the tortoise-shell palate plates by means of rivets, the porcelain gum attached to the teeth in most instances extending sufficiently high up to prevent the color of the shell being observable externally. The operator has now merely to attach his swivel and springs and the set is ready for insertion in his patient's mouth.

Throughout the delivery of the lecturer, Mr. H. was listened to with great attention, and at its conclusion, offered to explain any part of his process to any person interested, which we understand many availed themselves of. J. R.

## Figures and Tables

**Figure f1:**